# Supplementary material for: Mechanism of mitoribosomal small subunit biogenesis and preinitiation
Source: Nature. 2022 Jun 8;606(7914):603–8. doi: 10.1038/s41586-022-04795-x (PMC9200640; doi:10.1038/s41586-022-04795-x)
Supplement: Supplementary file 1 — This file contains Supplementary Figures 1–8; Supplementary Tables 1–4 and legends for Supplementary Videos 1–4 [file 41586_2022_4795_MOESM1_ESM.pdf]

nature portfolio

https://doi.org/10.1038/s41586-022-04795-x

Supplementary information

Mechanism of mitoribosomal small subunit biogenesis and preinitiation

In the format provided by the authors and unedited

Mechanism of mitoribosomal small subunit biogenesis and preinitiation

Yuzuru Itoh[1,9], Anas Khawaja[2,3,9], Ivan Laptev[4,5,6], Miriam Cipullo[2,3], Ilian Atanassov[7], Petr Sergiev[4,5,6,8], Joanna Rorbach[2,3*], Alexey Amunts[1*]

[1] Science for Life Laboratory, Department of Biochemistry and Biophysics, Stockholm University, 17165 Solna, Sweden.
[2] Department of Medical Biochemistry and Biophysics, Karolinska Institute, 17177 Stockholm, Sweden.
[3] Max Planck Institute for Biology of Ageing-Karolinska Institutet Laboratory, Karolinska Institutet, Stockholm 17165, Sweden.
[4] Center of Life Sciences, Skolkovo Institute of Science and Technology, Skolkovo, Moscow 143028, Russia.
[5] Department of Chemistry, Lomonosov Moscow State University, Moscow 119992, Russia.
[6] Belozersky Institute of Physico-Chemical Biology, Lomonosov Moscow State University, Moscow 119992, Russia.
[7] Proteomics Core Facility, Max-Planck-Institute for Biology of Ageing, Joseph-Stelzmann-Str. 9b, 50931 Cologne, Germany
[8] Institute of Functional Genomics, Lomonosov Moscow State University, Moscow 119992, Russia.
[9] These authors contributed equally

SUPPLEMENTARY INFORMATION

<u>Table of content:</u>

SI Figure 1. Cryo-EM processing overview for preSSU-1 and preSSU-2 states.

SI Figure 2. Cryo-EM processing overview for preSSU-3, preSSU-4, PIC-1, IC states.

SI Figure 3. Cryo-EM processing overview for preSSU-3, preSSU-4, PIC-1, IC and LSU states.

SI Figure 4. Full-length blots from figure 3d-e and extended data fig 11a.

SI Figure 5. Full-length sucrose gradient blots from figure 3e.

SI Figure 6. Full-length sucrose gradient blots from extended data fig 10b-c.

SI Figure 7. Western blot and sucrose gradient: HEK293T and mtIF3 KO.

SI Figure 8. Full-length blots from SI figure 7.

Supplementary Table 1. Cryo-EM statistics for preSSU-1 and preSSU-2.

Supplementary Table 2. Cryo-EM statistics for preSSU-3, preSSU-4, PIC-1, IC, LSU.

Supplementary Table 3. The positions/numbering of mouse 12S rRNA corresponding to that of human 12S rRNA.

Supplementary Table 4. List of guide RNAs and antibodies.

SI Video 1. Density maps and models of the SSU assembly states. Overall refined maps, combined local-masked refined maps, and models are shown for each of the states.

SI Video 2. States preSSU-1 to preSSU-3. Transition from preSSU-1 through preSSU-2 to preSSU-3, and RBFA conformational change.

SI Video 3. States preSSU-3a to preSSU-3c. Transition from preSSU-3a through preSSU-3b to preSSU-3c, and rRNA modifications.

SI Video 4. States preSSU-3 to PIC-1. Transition from preSSU-3 through PIC-1 to initiation complex with mRNA, fMet-tRNA(Met) with f5C modification, and mtIF2.

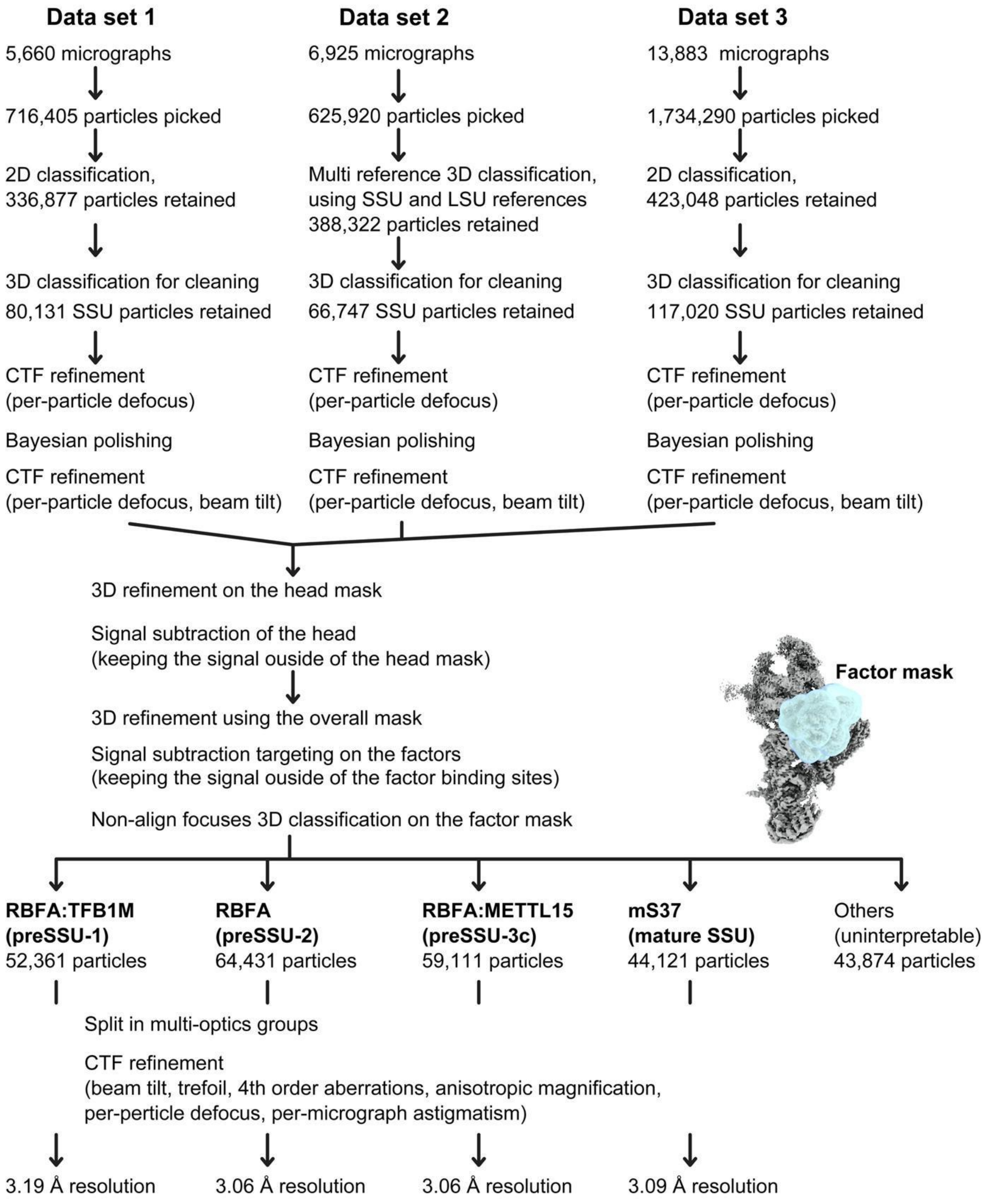

SI Figure 1. Cryo-EM processing overview for preSSU-1 and preSSU-2 states.

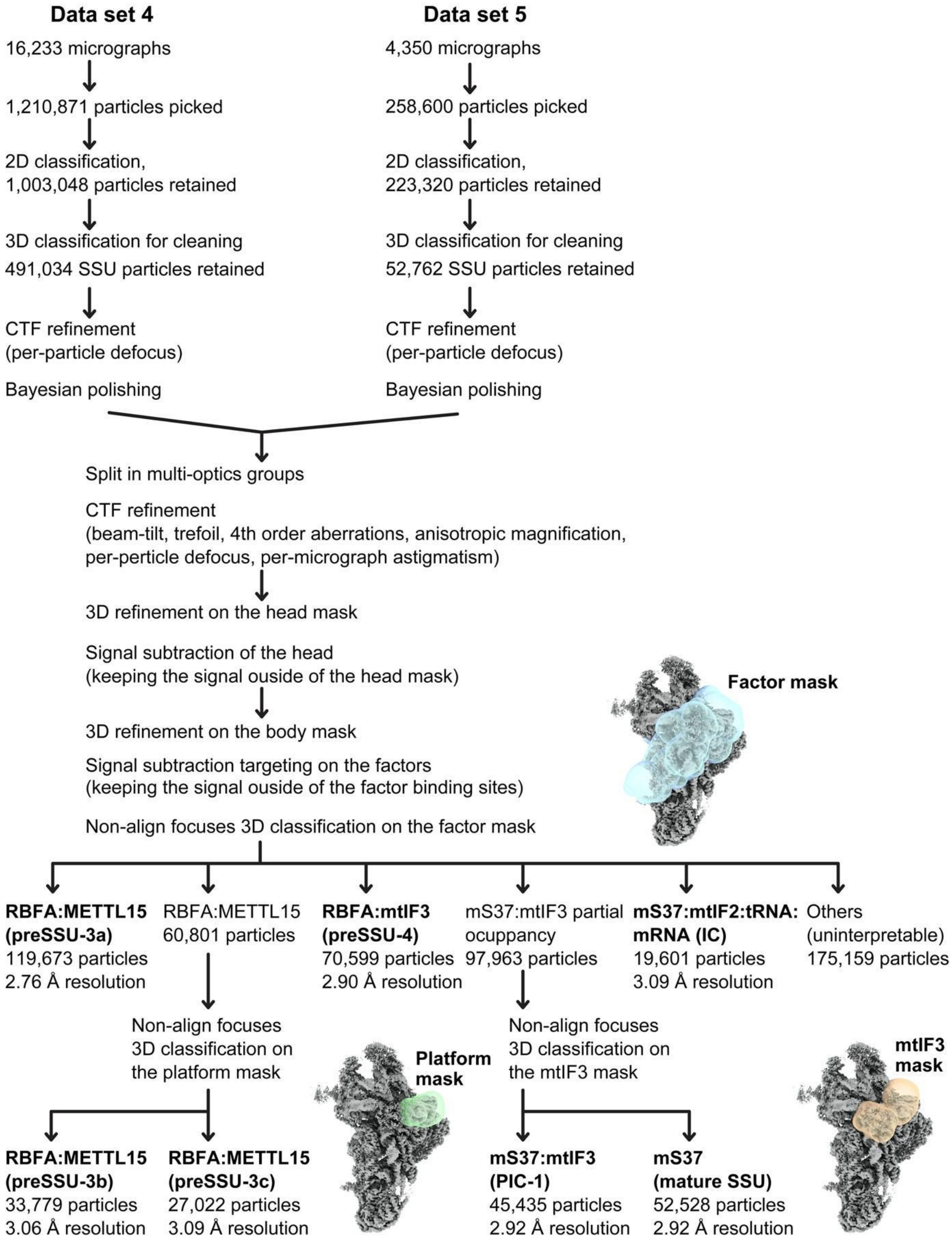

SI Figure 2. Cryo-EM processing overview for preSSU-3, preSSU-4, PIC-1, IC states.

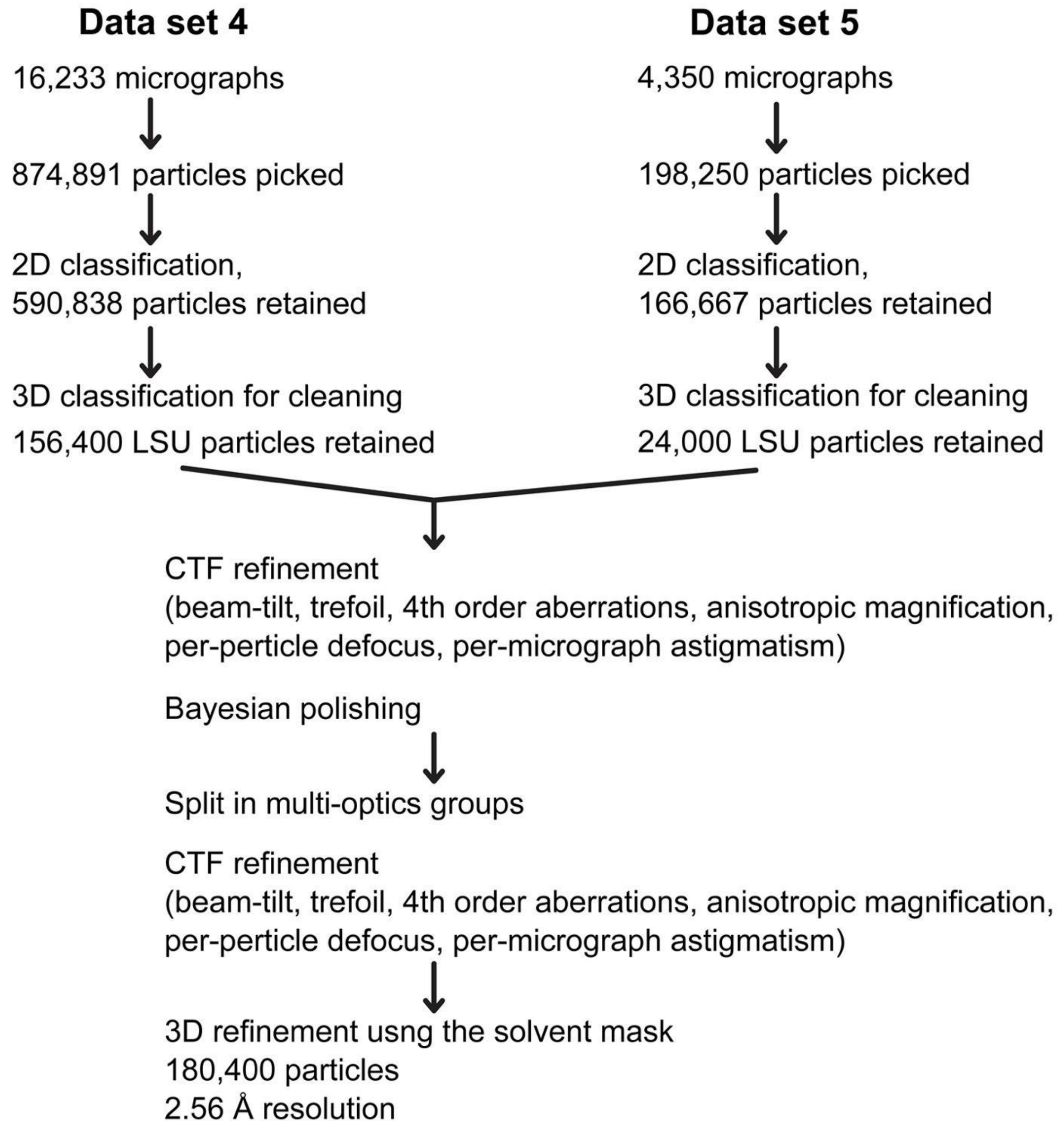

SI Figure 3. Cryo-EM processing overview for LSU.

Figure 3d. autoradiograph

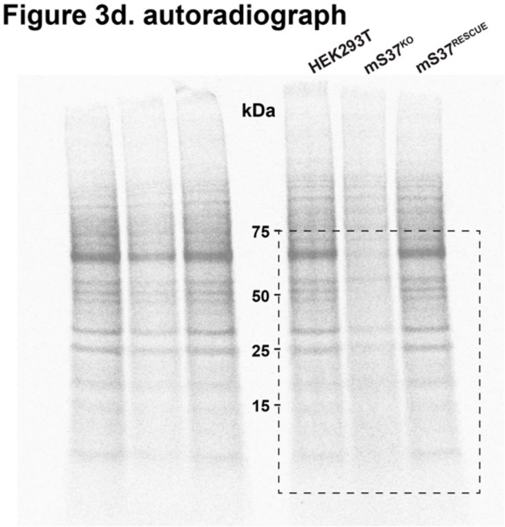

Figure 3d. loading control

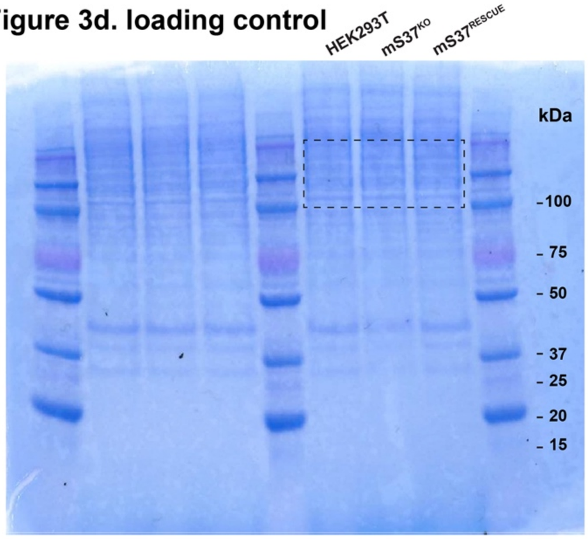

Figure 3e (A) and Extended Data Figure 11a (B)

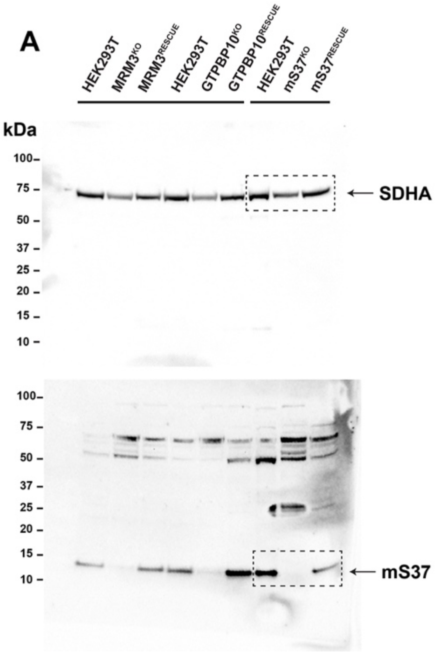

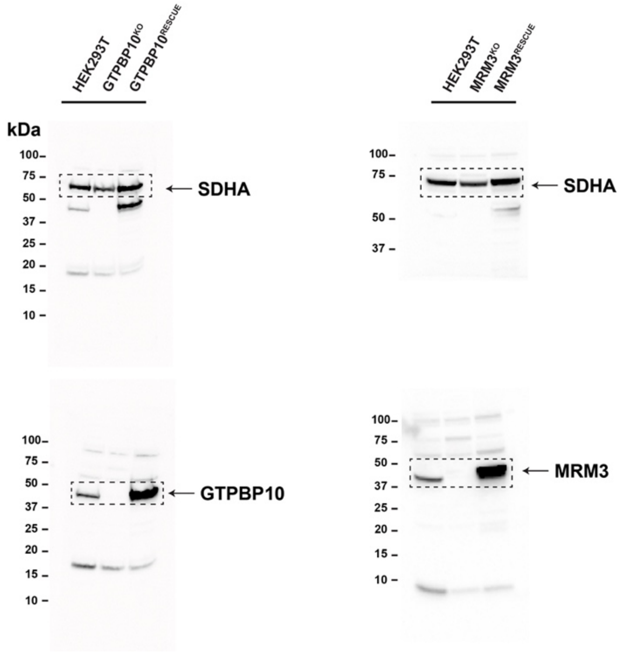

Figure 3e (B) and Extended Data Fig. 11a (A)

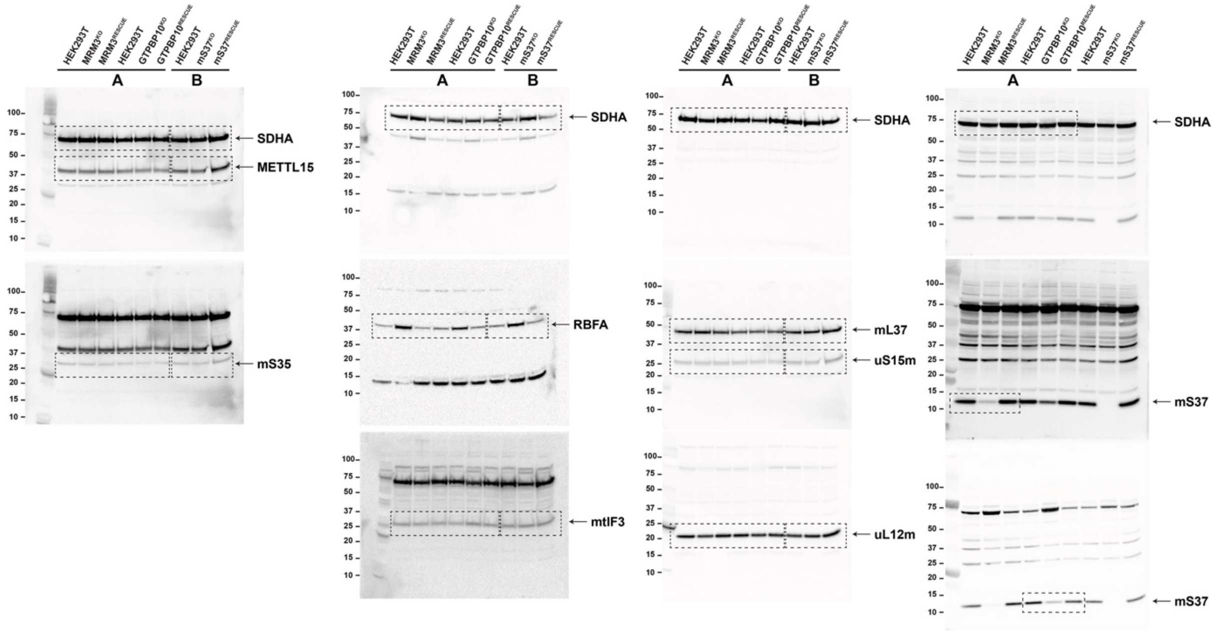

SI Figure 4. Full-length autoradiograph, loading control gel and western blots from figure 3d, 3e and Extended Data Fig 11a.

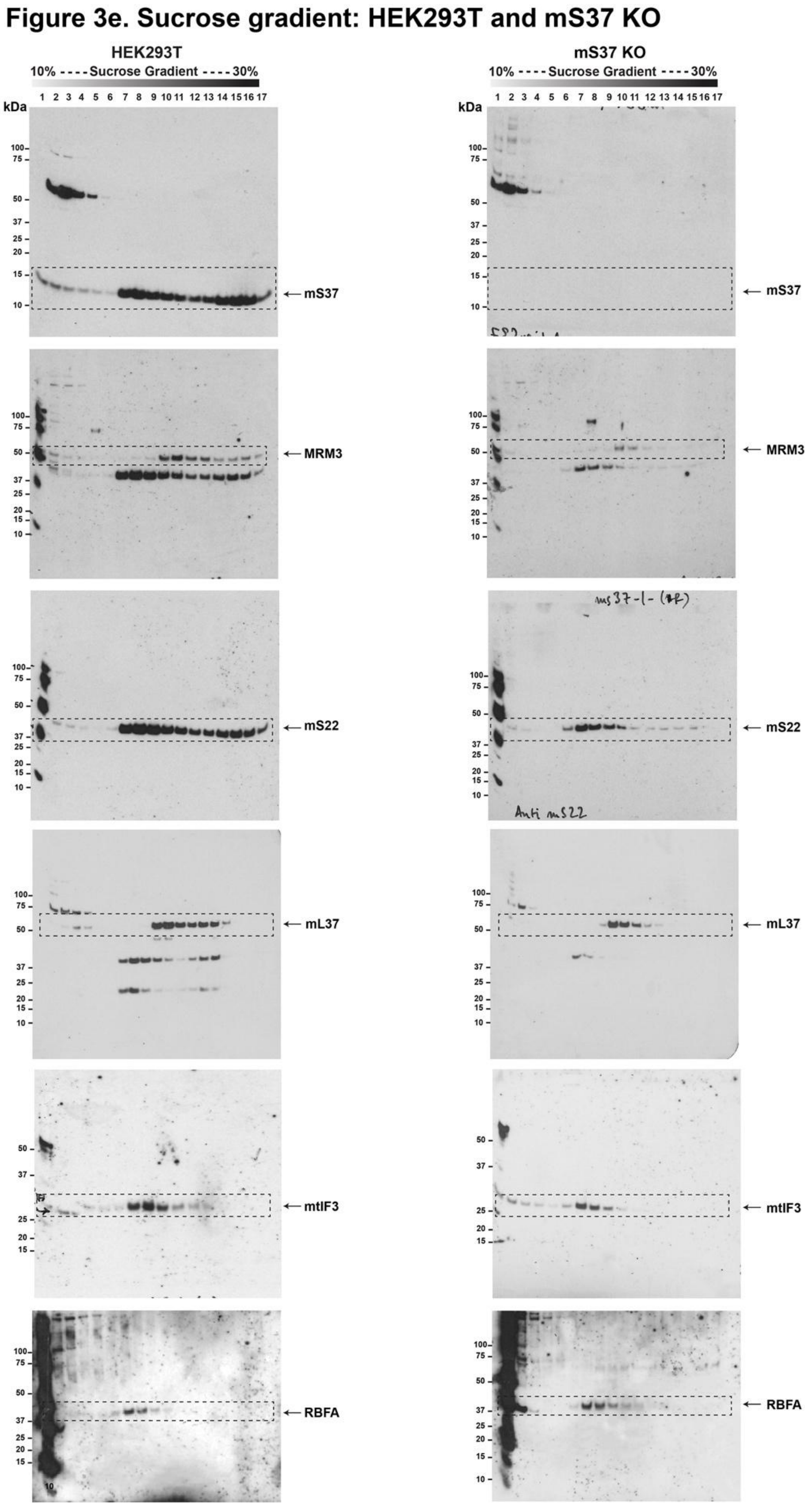

SI Figure 5. Full-length sucrose gradients blots from figure 3b.

Extended Data Fig. 10b. Autoradiograph

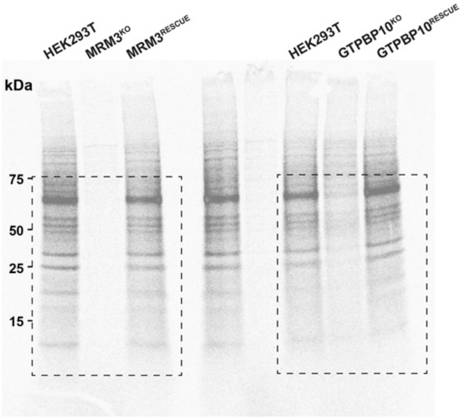

Extended Data Fig. 10b. Loading control

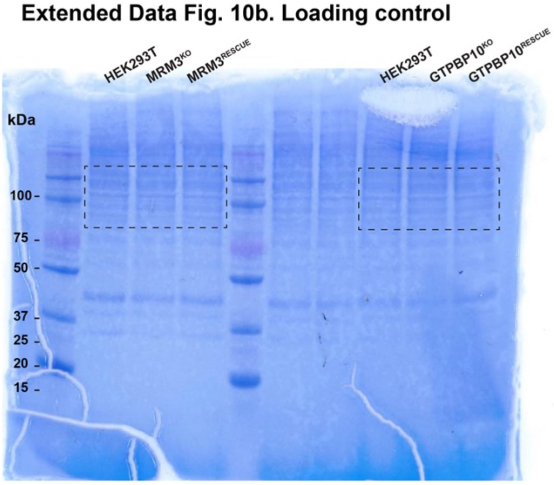

Extended Data Fig. 10c.
Sucrose gradient: HEK293T, MRM3 KO, GTPBP10 KO

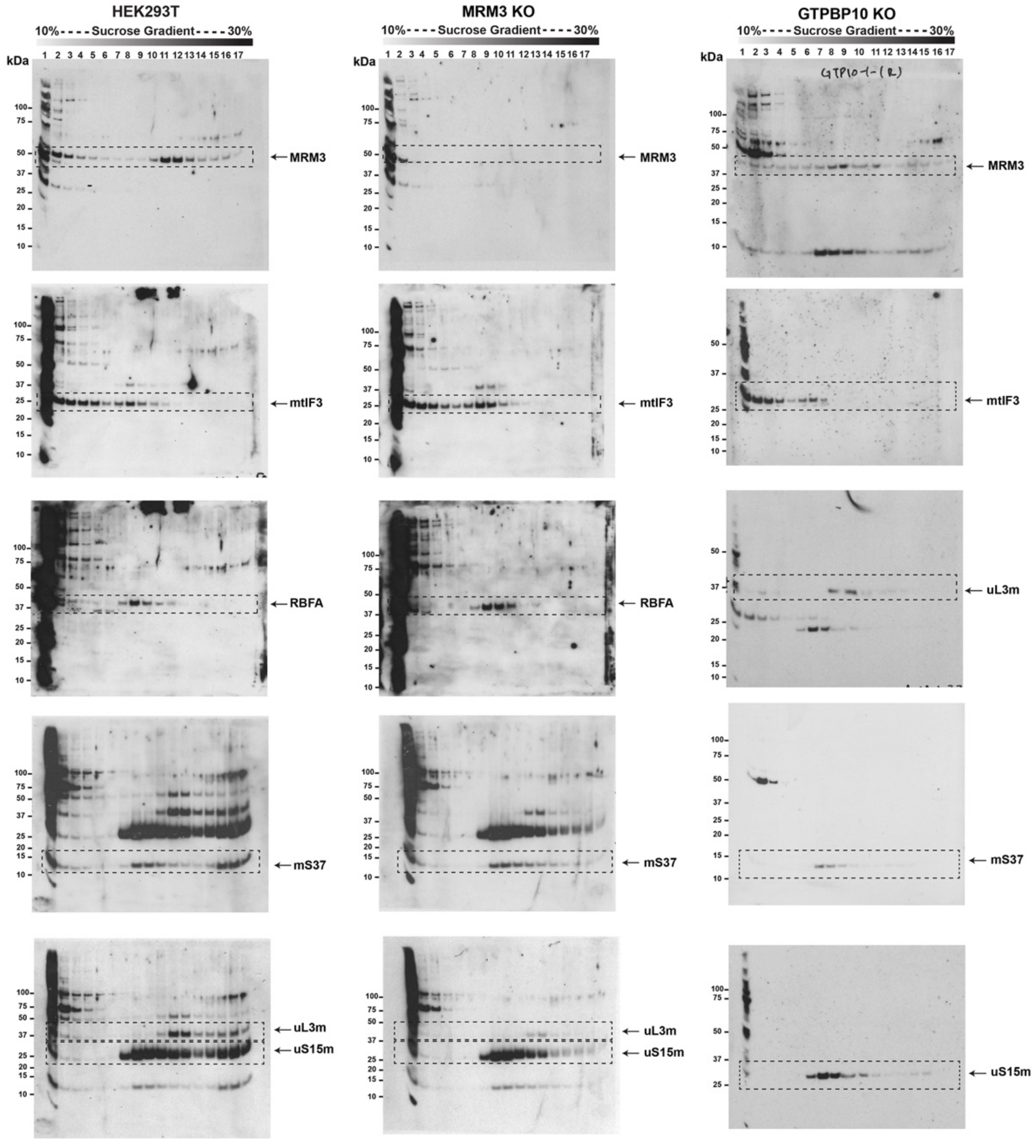

SI Figure 6. Full-length autoradiograph, loading control gel and sucrose gradient blots from Extended Data Fig. 10b,c.

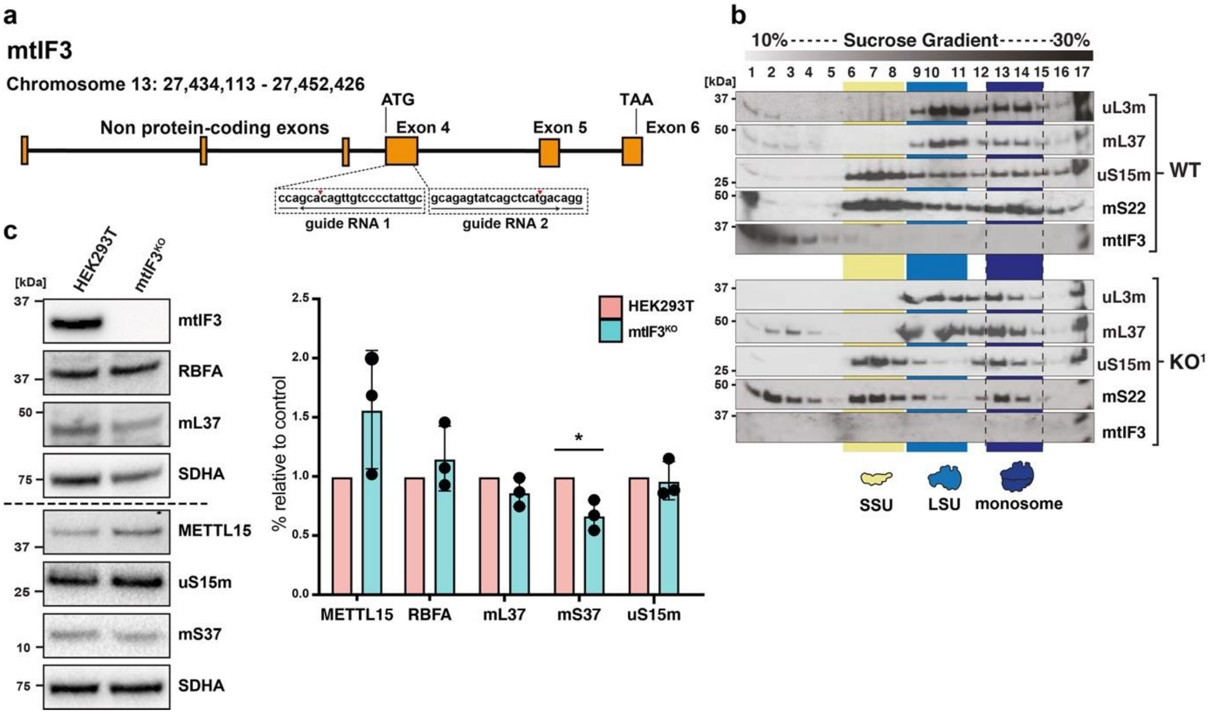

SI Figure 7. Western blot and sucrose gradient: HEK293T and mtIF3 KO.

a, A mtIF3 knockout cell line was generated. **b,** A monosome fraction is not majorly affected in mtIF3 knockout. **c,** A mild reduction of mS37 steady-state levels is consistent with the finding that mtIF3 binds before mS37. Steady-state levels of mitoribosomal proteins and assembly factors in the WT HEK293 and mtIF3KO were analyzed by immunoblotting; SDHA was used as a loading control (left panel). Quantifications of the steady-state levels were performed from three biologically independent experiments (right panel). Data were expressed as mean ± S.D; Student's two-tailed t-test was used (*$p < 0.05$).

SI Figure 7b. Sucrose gradient: HEK293T and mtIF3KO

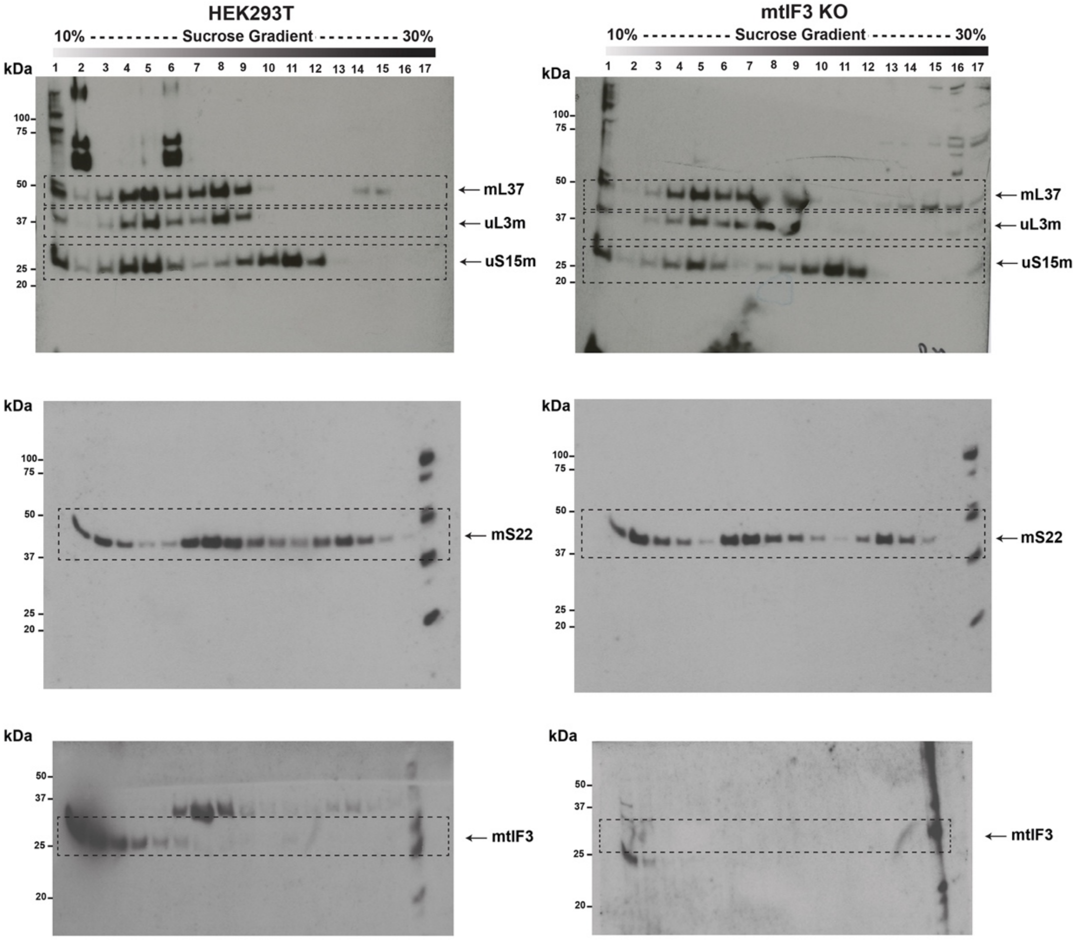

SI Figure 7c. Western blots

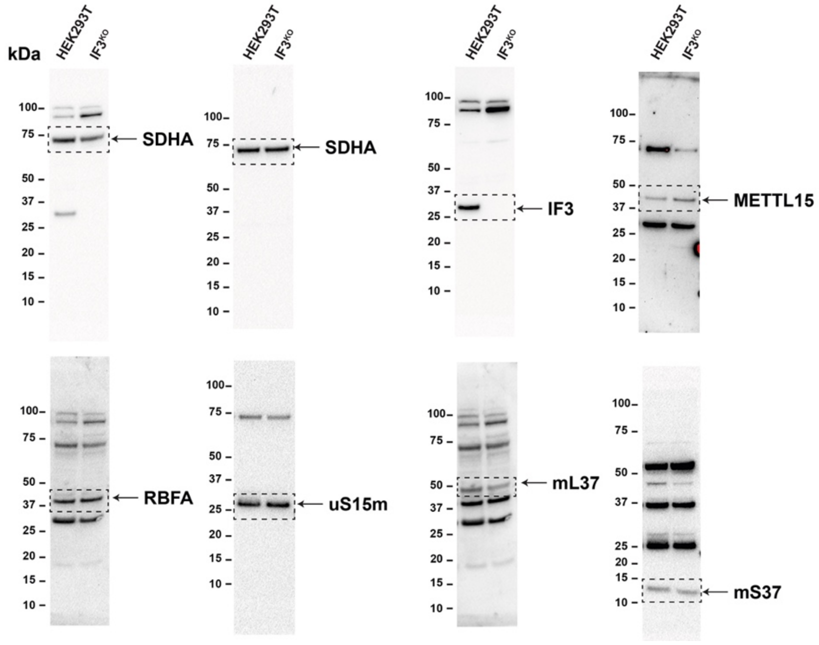

SI Figure 8. Full-length western blot and sucrose gradient: HEK293T and mtIF3 KO, related to Supplementary Information Fig. 7.

	**preSSU-1 (PDB: 7PNT) (EMD-13551)**	**preSSU-2 (PDB: 7PNU) (EMD-13552)**	**preSSU-3c (PDB: 7PNV) (EMD-13553)**	**Mature SSU (PDB: 7PNW) (EMD-13554)**
**Data collection and Processing**				
Microscope	Titan Krios	Titan Krios	Titan Krios	Titan Krios
Voltage (kV)	300	300	300	300
Camera	K2 Summit	K2 Summit	K2 Summit	K2 Summit
Magnification	165,000	165,000	165,000	165,000
Pixel size at detector (Å/pixel)	0.83	0.83	0.83	0.83
Total electron exposure ($e^-/Å^2$)	30–31	30–31	30–31	30–31
Exposure rate (e^-/pixel/sec)	5.0–5.3	5.0–5.3	5.0–5.3	5.0–5.3
Number of frames	20	20	20	20
Defocus range (μm)	−0.8 to −3.6	−0.8 to −3.6	−0.8 to −3.6	−0.8 to −3.6
Energy filter slit width (V)	20	20	20	20
Micrographs collected (no.)	26,468	26,468	26,468	26,468
Final particles (no.)	52,361	64,431	59,111	44,121
Point group	C_1	C_1	C_1	C_1
Resolution (global, Å) FSC 0.143 (masked) (Overall/ shoulder/ platform/ back/ tail/ head-AP/ head-PE/ mS39/ factor)	3.19/ 2.93/ 3.06/ 2.93/ 2.93/ 3.06/ 3.09/ 3.24/ 2.95 (RBFA)/ 3.00 ((TFB1M)	3.06/ 2.86/ 2.93/ 2.87/ 2.89/ 2.93/ 2.93/ 3.24/ 2.93 (RBFA)	3.06/ 2.81/ 2.95/ 2.87/ 3.19/ 2.91/ 2.93/ 2.89/ 2.89 (RBFA)/ 2.91 (METTL15)	3.09/ 2.83/ 2.93/ 2.91/ 2.95/ 2.97/ 3.00/ 3.29
Map-sharpening *B* factor ($Å^2$) (Overall/ shoulder/ platform/ back/ tail/ head-AP/ head-PE/ mS39/ factor)	−51/ −50/ −49/ −37/ −49/ −55/ −50/ −82/ −56 (RBFA)/ −58 (TFB1M)	−54/ −45/ −50/ −48/ −47/ −48/ −48/ −71/ −56 (RBFA)	−41/ −44/ −41/ −45/ −64/ −41/ −46/ −46/ −46 (RBFA)/ −55 (METTL15)	−40/ −42/ −41/ −45/ −47/ −43/ −44/ −69
**Model composition**				
Atoms (non-hydrogen/ hydrogen)	68,913/ 59,549	67,976/ 57,668	71,078/ 60,889	67,911/ 57,690
Chains (RNA/ protein)	1/ 31	1/ 30	1/ 31	1/ 30
RNA residues (non-modified/ modified)	909/ 0	941 / 0	947/ 4	950 /4
Protein residues (non-modified/ modified)	6,038/ 2	5,765 / 2	6,143 / 2	5,744 / 3
Metal ions (Mg^{2+}/ K^+/ Zn^{2+})	34/ 7/ 1	42/ 7/ 1	49/ 13/ 1	57/ 10/ 1
Ligands (2Fe-2S /ATP/ SAH)	2/ 1/ 0	2/ 1/ 0	2/ 1/ 1	2/ 1/ 0
Waters	470	1,017	868	783
**Model Refinement**				
Model-Map CC (CC_{mask}/ CC_{box}/ CC_{peaks}/ CC_{volume})	0.76/ 0.76/ 0.68/ 0.74	0.79/ 0.77/ 0.71/ 0.77	0.79/ 0.78/ 0.71/ 0.77	0.80/ 0.79/ 0.72/ 0.78
Resolution (Å) by model-to-map FSC, threshold 0.50 (masked/ unmasked)	2.99/ 3.00	2.87/ 2.87	2.87/ 2.87	2.88/ 2.89
Average *B* factor ($Å^2$) (RNA/ protein/ metal ion and ligand/ water)	34/ 21/ 18/ 13	25/ 18/ 13/ 9.4	16/ 15/ 8.3/ 5.7	23/ 18/ 17/ 12
R.m.s. deviations, bond lengths (Å)/ bond angles (°)	0.002/ 0.422	0.002/ 0.425	0.002/ 0.433	0.002/ 0.422
**Validation**				
MolProbity score	0.89	0.90	0.86	0.87
CaBLAM outliers (%)	0.63	0.62	0.67	0.61
Clash score	1.49	1.54	1.32	1.37
Rotamer outliers (%)	0.02	0.02	0.02	0.02
C_β deviations	0	0	0	0
EMRinger score	3.80	4.11	3.83	3.89
Ramachandran plot (%) (Favored/ allowed/ disallowed)	98.51/ 1.46/ 0.03	98.52/ 1.44/ 0.04	98.45/ 1.53/ 0.02	98.54/ 1.45/ 0.02

Supplementary Table 1. Cryo-EM statistics for preSSU-1 and preSSU-2.

Data collection, processing, model refinement and validation statistics for states preSSU-1 and preSSU-2 states.

	**preSSU-3a (PDB: 7PNX) (EMD-13555)**	**preSSU-3b (PDB: 7PNY) (EMD-13556)**	**preSSU-3c (PDB: 7PNZ) (EMD-13557)**	**preSSU-4 (PDB: 7PO0) (EMD-13558)**	**PIC-1 (PDB: 7PO1) (EMD-13559)**	**IC (PDB: 7PO2) (EMD-13560)**	**Mature SSU (PDB: 7PO3) (EMD-13561)**	**preLSU (PDB: 7PO4) (EMD-13562)**
**Data collection and Processing**								
Microscope	Titan Krios	Titan Krios	Titan Krios	Titan Krios	Titan Krios	Titan Krios	Titan Krios	Titan Krios
Voltage (kV)	300	300	300	300	300	300	300	300
Camera	K2 Summit	K2 Summit	K2 Summit	K2 Summit	K2 Summit	K2 Summit	K2 Summit	K2 Summit
Magnification	165,000	165,000	165,000	165,000	165,000	165,000	165,000	165,000
Pixel size at detector (Å/pixel)	0.81	0.81	0.81	0.81	0.81	0.81	0.81	0.81
Total electron exposure (e^-/Å^2)	31–32	31–32	31–32	31–32	31–32	31–32	31–32	31–32
Exposure rate (e^-/pixel/sec)	4.1–5.9	4.1–5.9	4.1–5.9	4.1–5.9	4.1–5.9	4.1–5.9	4.1–5.9	4.1–5.9
Number of frames	20	20	20	20	20	20	20	20
Defocus range (μm)	−1.0 to −3.8	−1.0 to −3.8	−1.0 to −3.8	−1.0 to −3.8	−1.0 to −3.8	−1.0 to −3.8	−1.0 to −3.8	−1.0 to −3.8
Energy filter slit width (V)	20	20	20	20	20	20	20	20
Micrographs collected (no.)	20,583	20,583	20,583	20,583	20,583	20,583	20,583	20,583
Final particles (no.)	119,673	33,779	27,022	70,599	45,435	19,601	52,528	180,400
Point group	C_1	C_1	C_1	C_1	C_1	C_1	C_1	C_1
Resolution (global, Å) FSC 0.143 (masked) (Overall/ shoulder/ platform/ back/ tail/ head-AP/ head-PE/ mS39/ factor)	2.76/ 2.49/ 2.59/ 2.56/ 2.72/ 2.57/ 2.74/ 2.86/ 2.59 (RBFA)/ 2.64 (METTL15)	3.06/ 2.78/ 2.92/ 2.90/ 3.09/ 2.90/ 2.99/ 3.27/ 2.92 (RBFA)/ 2.95 (METTL15)	3.09/ 2.86/ 2.95/ 2.92/ 3.11/ 2.95/ 3.04/ 3.29/ 2.97 (RBFA)/ 2.99 (METTL15)	2.90/ 2.63/ 2.72/ 2.74/ 2.86/ 2.72/ 2.86/ 3.11/ 2.67 (RBFA)/ 2.68 (IF3)	2.92/ 2.70/ 2.76/ 2.72/ 2.92/ 2.82/ 2.97/ 3.11/ 2.70 (IF3)	3.09/ 2.90/ 2.92/ 2.92/ 3.19/ 2.92/ 3.06/ 3.32/ 2.97 (IF2)	2.92/ 2.67/ 2.70/ 2.72/ 2.86/ 2.76/ 2.90/ 3.19	2.56/ 2.52 (Body)/ 2.76 (CP)/ 2.98 (L10-L7/L12)/ 2.60 (MALSU1-ACP)
Map-sharpening *B* factor (Å^2) (Overall/ shoulder/ platform/ back/ tail/ head-AP/ head-PE/ mS39/ factor)	−42/ −38/ −41/ −46/ −47/ −41/ −54/ −61/ −44 (RBFA)/ −53 (METTL15)	−46/ −39/ −42/ −39/ −49/ −43/ −45/ −73/ −51 (RBFA)/ −50 (METTL15)	−46/ −37/ −44/ −36/ −64/ −41/ −48/ −61/ −47 (RBFA)/ −40 (METTL15)	−42/ −40/ −42/ −47/ −51/ −40/ −44/ −67/ −47 (RBFA)/ −47 (IF3)	−44/ −35/ −37/ −42/ −49/ −44/ −52/ −57/ −40 (IF3)	−44/ −31/ −37/ −43/ −58/ −37/ −37/ −67/ −37 (IF2)	−40/ −34/ −38/ −38/ −51/ −42/ −58/ −69	−40/ −38 (Body)/ −51 (CP)/ −59 (L10-L7/L12)/ −54 (MALSU1-ACP)/
**Model composition**								
Atoms (non-hydrogen/ hydrogen)	72,634/ 61,133	71,215/ 60,982	71,258/ 60,982	70,406/ 59,872	69,876/ 59,325	75,175/ 63,995	68,637/ 57,624	100,401/ 85,962
Chains (RNA/ protein)	1/ 31	1/ 31	1/ 31	1/ 31	1/ 31	3/ 32	1/ 30	2/ 58
RNA residues (non-modified/ modified)	946/ 5	946 / 5	946/ 5	946/ 5	950/ 5	1,051/ 6	950/ 5	1,214/ 3
Protein residues (non-modified/ modified)	6,155/ 2	6137/ 2	6,137/ 2	5,994/ 2	5,913/ 3	6,371/ 3	5,710/ 3	8,822/ 4
Metal ions (Mg^{2+}/ K^+/ Zn^{2+})	64/ 16/ 1	61/ 20/ 1	61/ 17/ 1	65/ 17/ 1	71/ 20/ 1	72/ 20/ 1	64/ 21/ 1	35/ 8/ 1
Ligands (2Fe-2S /ATP/ GDP/ NAD/ other)	2/ 1/ 1/ 1/ 1 (SAH)	2/ 1/ 1/ 1/ 1 (SAH)	2/ 1/ 1/ 1/ 1 (SAH)	2/ 1/ 1/ 1/ 0	2/ 1/ 1/ 0/ 0	2/ 1/ 1/ 0/ 1 (GTP)/ 1 (fMet)	2/ 1/ 1/ 0/ 0	1/ 0/ 0/ 0/ 1 (*S*-dodecanoyl-4′-PP)
Waters	2,156	882	928	1,166	1,173	807	1,578	2,543
**Model Refinement**								
Model-Map CC (CC_{mask}/ CC_{box}/ CC_{peaks}/ CC_{volume})	0.85/ 0.80/ 0.75/ 0.82	0.79/ 0.78/ 0.71/ 0.77	0.79/ 0.78/ 0.71/ 0.77	0.82/ 0.79/ 0.74/ 0.80	0.84/ 0.81/ 0.76/ 0.81	0.79/ 0.78/ 0.71/ 0.77	0.85/ 0.81/ 0.77/ 0.83	0.83/ 0.77/ 0.72/ 0.80
Resolution (Å) by model-to-map FSC, threshold 0.50 (masked/ unmasked)	2.51/ 2.52	2.90/ 2.90	2.90/ 2.91	2.70/ 2.75	2.73/ 2.74	2.93/ 2.94	2.69/ 2.69	2.40/ 2.40
Average *B* factor (Å^2) (RNA/ protein/ metal ion and ligand/ water)	30/ 28/ 19/ 20	22/ 20/ 14/ 13	23/ 22/ 16/ 13	19/ 19/ 13/ 13	26/ 22/ 15/ 12	26/ 23/ 23 17	26/ 23/ 15/ 11	43/ 28/ 33/ 14
R.m.s. deviations, bond lengths (Å)/ bond angles (°)	0.002/ 0.416	0.002/ 0.407	0.002/ 0.408	0.002/ 0.408	0.002/ 0.402	0.002/ 0.406	0.002/ 0.406	0.002/ 0.465
**Validation**								
MolProbity score	0.96	1.00	0.99	0.94	0.94	0.94	0.96	1.00
CaBLAM outliers (%)	0.67	0.74	0.72	0.70	0.67	0.64	0.68	0.67
Clash score	1.97	2.24	2.19	1.81	1.79	1.81	1.91	2.20
Rotamer outliers (%)	0.04	0.06	0.06	0.02	0.06	0.05	0.04	0.03
C_β deviations	0	0	0	0	0	0	0	0
Ramachandran plot (%) (Favored/ allowed/ disallowed)	98.74/ 1.24/ 0.02	98.72/ 1.26/ 0.02	98.77/ 1.19/ 0.03	98.81/ 1.17/ 0.02	98.92/ 1.06/ 0.02	98.81/ 1.17/ 0.02	98.97/ 1.01/ 0.02	98.40/ 1.60/ 0.00
EMRinger score	5.10	4.47	4.20	4.89	4.84	4.44	5.07	5.09

Supplementary Table 2. Cryo-EM statistics for preSSU-3, preSSU-4, PIC-1, IC, LSU.

Data collection, processing, model refinement and validation statistics for states preSSU-3, preSSU-4, PIC-1, IC, and LSU.

	H. sapiens	*M. musculus*
5′-end	648	1
h23	974-1014	321-363
h24	1057-1098	406-447
h27	1138-1165	488-515
h28	1174-1192<br>1462-1480	524-541<br>816-834
h28-h44 linker	1481-1487	835-841
h44	1488-1562	842-916
h44-h45 linker	1563-1571	917-925
h45	1572-1593	926-947
3′-end	1594-1602	948-956
m^5U (by TRMT2B)	1076	425
m^4C (by METTL15)	1486	840
m^5C (by NSUN4)	1488	842
m^6_2A (by TFB1M)	1583	937
m^6_2A (by TFB1M)	1584	938

Supplementary Table 3. The positions/numbering of mouse 12S rRNA corresponding to that of human 12S rRNA.

Antibody	Company	Catalog no.	Dilutions
SDHA	Abcam	ab14715	1:2000
C18orf22/RBFA	Abcam	ab224741	1:1000
METTL15	Merck	HPA039885	1:1000
CHCHD1/mS37	ThermoFisher Scientific	**PA5-58635**	**1:500**
mtIF3	Proteintech Group	14219-1-AP	1:1000
GTPBP10	Merck	HPA021076	1:500
RNMTL1/MRM3	Merck	HPA023292	1:1000
uS15m	Proteintech Group	17006-1-AP	1:2000
mS22	Proteintech Group	10984-1-AP	1:2000
mS35	Proteintech Group	16457-1-AP	1:2000
uL3m	Merck	HPA043665	1:1000
uL12m	Merck	HPA022853	1:2000
mL37	Merck	HPA025826	1:2000
HRP secondary anti-rabbit	GE Healthcare	NA9340V	1:2000
HRP secondary anti-mouse	GE Healthcare	NA9310V	1:2000

**gRNA for mS37 exon 1**	**Sequence 5′ to 3′**
gRNA 1 top	CACCGGGTTTATTGGGCTTCAGCAC
gRNA 1 bottom	AAACGTGCTGAAGCCCAATAAACCC
gRNA 2 top	CACCGCCGCGTCGGGGAGCGGCGCC
gRNA 2 bottom	AAACGGCGCCGCTCCCCGACGCGGC
**gRNA for MRM3 exon 1**	
gRNA 1 top	CACCGCTACGATAAAGCTTATCCCG
gRNA 1 bottom	AAACCGGGATAAGCTTTATCGTAGC
gRNA 2 top	CACCGTGTTCCACCACCTCTCCGGA
gRNA 2 bottom	AAACTCCGGAGAGGTGGTGGAACAC
**gRNA for GTPBP10 exon 1**	
gRNA 1 top	CACCGCCAGCCACAAACCGTTTCCG
gRNA 1 bottom	AAACCGGAAACGGTTTGTGGCTGGC
gRNA 2 top	CACCGACGAGGATAACCCATTCCAC
gRNA 2 bottom	AAACGTGGAATGGGTTATCCTCGTC

Supplementary Table 4. List of guide RNAs and antibodies.
