# Supplementary material for: Mechanism of mitoribosomal small subunit biogenesis and preinitiation
Source: Nature. 2022 Jun 8;606(7914):603–8. doi: 10.1038/s41586-022-04795-x (PMC9200640; doi:10.1038/s41586-022-04795-x)
Supplement: Supplementary file 2 — Reporting Summary [file 41586_2022_4795_MOESM2_ESM.pdf]

nature portfolio

Corresponding author(s): Joanna Rorbach, Alexey Amunts

Last updated by author(s): Apr 10, 2022

Reporting Summary

Nature Portfolio wishes to improve the reproducibility of the work that we publish. This form provides structure for consistency and transparency in reporting. For further information on Nature Portfolio policies, see our Editorial Policies and the Editorial Policy Checklist.

Statistics

For all statistical analyses, confirm that the following items are present in the figure legend, table legend, main text, or Methods section.

n/a	Confirmed	
☐	☒	The exact sample size (*n*) for each experimental group/condition, given as a discrete number and unit of measurement
☐	☒	A statement on whether measurements were taken from distinct samples or whether the same sample was measured repeatedly
☐	☒	The statistical test(s) used AND whether they are one- or two-sided<br>*Only common tests should be described solely by name; describe more complex techniques in the Methods section.*
☒	☐	A description of all covariates tested
☒	☐	A description of any assumptions or corrections, such as tests of normality and adjustment for multiple comparisons
☐	☒	A full description of the statistical parameters including central tendency (e.g. means) or other basic estimates (e.g. regression coefficient) AND variation (e.g. standard deviation) or associated estimates of uncertainty (e.g. confidence intervals)
☐	☒	For null hypothesis testing, the test statistic (e.g. *F*, *t*, *r*) with confidence intervals, effect sizes, degrees of freedom and *P* value noted<br>*Give P values as exact values whenever suitable.*
☒	☐	For Bayesian analysis, information on the choice of priors and Markov chain Monte Carlo settings
☒	☐	For hierarchical and complex designs, identification of the appropriate level for tests and full reporting of outcomes
☒	☐	Estimates of effect sizes (e.g. Cohen's *d*, Pearson's *r*), indicating how they were calculated

Our web collection on statistics for biologists contains articles on many of the points above.

Software and code

Policy information about availability of computer code

Data collection	The datasets were collected EPU 1.9 software on FEI Titan Krios (FEI/Thermofischer) transmission electron microscope operated at 300 keV with a slit width of 20 eV on a GIF quantum energy filter (Gatan). A K2 Summit detector (Gatan) was used at a pixel size of 0.81 or 0.83 Å (magnification of 165,000x) with a dose of 30-32 electrons/Å2 fractionated over 20 frames. A defocus range of 0.8 to 3.8 μm was used. A total of 5 datasets were recorded and kept.
Data analysis	Movie frames were aligned and averaged by global and local motion corrections by the program MotionCor2. Contrast transfer function (CTF) parameters were estimated by GCTF. Particles were picked by Gautomatch and 2D classified by RELION 3.0. The models were manually built with Coot 0.9 and stereochemical refinement was performed using phenix.real_space_refine in the PHENIX 1.17.1 suite. Models were predicted with AlphaFold2. The final model was validated using MolProbity. Figures were prepared with UCSF Chimera X 0.91.

For manuscripts utilizing custom algorithms or software that are central to the research but not yet described in published literature, software must be made available to editors and reviewers. We strongly encourage code deposition in a community repository (e.g. GitHub). See the Nature Portfolio guidelines for submitting code & software for further information.

Data

Policy information about availability of data

All manuscripts must include a data availability statement. This statement should provide the following information, where applicable:

- Accession codes, unique identifiers, or web links for publicly available datasets
- A description of any restrictions on data availability
- For clinical datasets or third party data, please ensure that the statement adheres to our policy

The atomic coordinates were deposited in the RCSB Protein Data Bank and Electron Microscopy Data bank under accession numbers: 7PNT and EMD-13551

(preSSU-1), 7PNU and EMD-13552 (preSSU-2), 7PNV and EMD-13553 (preSSU-3c), 7PNW and EMD-13554 (mature SSU), 7PNX and EMD-13555 (preSSU-3a), 7PNY and EMD-13556 (preSSU-3b), 7PNZ and EMD-13557 (preSSU-3c), 7PO0 and EMD-13558 (preSSU-4), 7PO1 and EMD-13559 (PIC-1), 7PO2 and EMD-13560 (IC), 7PO3 and EMD-135561 (mature SSU), 7PO4 and EMD-135562 (preLSU).

Field-specific reporting

Please select the one below that is the best fit for your research. If you are not sure, read the appropriate sections before making your selection.

☒ Life sciences ☐ Behavioural & social sciences ☐ Ecological, evolutionary & environmental sciences

For a reference copy of the document with all sections, see nature.com/documents/nr-reporting-summary-flat.pdf

Life sciences study design

All studies must disclose on these points even when the disclosure is negative.

Sample size	26,468 and 20,583 micrographs were analyzed for TRMT2B KO and MRM3 KO mitoribosomal structures, respectively. No statistical analyses has been performed. The number of cryo-EM particles in the single dataset collected was the number of particles available. No predetermined sample size was used for other experiments.
Data exclusions	Particles that were not mitoribosome were excluded in the analysis, since they cannot contribute to reconstruction.
Replication	All biochemical experiments that includes gradient purification, steady-state levels in the knock-outs and rescue cell lines were performed at least 3 times. All attempts in replication were successful. Similar cryo-EM structures were successfully obtained from preliminary datasets.
Randomization	Particle images were randomly assigned into half-sets to obtain gold-standard resolution estimates as described in the text.
Blinding	N/A to cryo-EM study; raw micrographs or particle images are not categorical data. Particles are randomly assigned into half-sets for image processing; hence no blinding is applicable.

Reporting for specific materials, systems and methods

We require information from authors about some types of materials, experimental systems and methods used in many studies. Here, indicate whether each material, system or method listed is relevant to your study. If you are not sure if a list item applies to your research, read the appropriate section before selecting a response.

Materials & experimental systems

n/a	Involved in the study	
☐	☒	Antibodies
☐	☒	Eukaryotic cell lines
☒	☐	Palaeontology and archaeology
☒	☐	Animals and other organisms
☒	☐	Human research participants
☒	☐	Clinical data
☒	☐	Dual use research of concern

Methods

n/a	Involved in the study	
☒	☐	ChIP-seq
☒	☐	Flow cytometry
☒	☐	MRI-based neuroimaging

Antibodies

Antibodies used	All antibodies used in this study are listed in detail in Supplementary Information Table 2.
Validation	All antibodies are commercially available and were commercially validated.

Eukaryotic cell lines

Policy information about cell lines

Cell line source(s)	Flp-In TREx 293 cell line was purchased from Thermofisher Scientific.<br>NS0 murine cell line (commercial source Merck 85110503) was obtained from the research facility "Vertebrate cell culture collection" of the Institute of Cytology of the Russian Academy of Sciences, St-Petersburg.
Authentication	Flp-In™ T-REx™ Cell Line (Catalog number: R78007) was purchased from Thermofisher Scientific. No authentication is required as these cells are Zeocin and Blasticidin resistant, in contrast to any other cell line.<br>Authentification of kinetic cell morhpology parameters was done for NS0 source cell line in the research facility "Vertebrate cell culture collection" of the Institute of Cytology of the Russian Academy of Sciences, St-Petersburg.

Mycoplasma contamination	Cell line tested negative for mycoplasma contamination.
Commonly misidentified lines (See ICLAC register)	No commonly misidentified cell lines were used in the study.
